# Supplementary material for: Exploring the Link between Social Support and Patient-Reported Outcomes in Chronic Obstructive Pulmonary Disease Patients: A Cross-Sectional Study in Primary Care
Source: Healthcare (Basel). 2024 Feb 25;12(5):544. doi: 10.3390/healthcare12050544 (PMC10930953; doi:10.3390/healthcare12050544)
Supplement: Supplementary file 1 [file healthcare-12-00544-s001.zip › healthcare-2877402-supplementary.pdf]

Table S1. Adjusted associations between perceived social support (continuous scales) and PROMs, estimated by linear regression models.

**Symptoms**	**N**	**MSPSS "significant other" (range 1-7)**		**MSPSS "family" (range 1-7)**		**MSPSS "friends" *(range 1-7)***		***MSPSS total (range 1-7)***	
		Beta (95%CI)	p-value	Beta (95%CI)	p-value	Beta (95%CI)	p-value	Beta (95%CI)	p-value
**CAT score**	163	-0.03 (-0.06, -0.01)	0.01	-0.03 (-0.06, -0.01)	0.01	-0.03 (-0.07, 0)	0.08	-0.03 (-0.06, -0.01)	0.01
CAT score≥10	163	-0.37 (-0.68, -0.06)	0.02	-0.37 (-0.68, -0.06)	0.02	-0.3 (-0.75, 0.14)	0.18	-0.35 (-0.67, -0.03)	0.03
**Daytime symptoms**									
**Fatigue**									
FSS (range 9-63)	161	-0.01 (-0.02, 0)	0.01	-0.02 (-0.03, 0)	0.01	-0.02 (-0.03, 0)	0.02	-0.02 (-0.03, 0)	0.01
FSS ≥ 36	161	-0.15 (-0.46, 0.16)	0.34	-0.25 (-0.56, 0.06)	0.11	-0.17 (-0.6, 0.27)	0.45	-0.19 (-0.51, 0.13)	0.24
**Daytime sleepiness**									
ESS (range 0-24)	160	-0.03 (-0.06, 0.01)	0.10	-0.03 (-0.06, 0)	0.07	-0.04 (-0.09, 0)	0.06	-0.03 (-0.07, 0)	0.05
ESS≥11	160	-0.18 (-0.6, 0.23)	0.39	-0.23 (-0.64, 0.18)	0.27	-0.32 (-0.91, 0.26)	0.28	-0.24 (-0.67, 0.18)	0.26
**Depressive symptoms**									
PHQ-9 (range 0-27)	163	-0.05 (-0.09, -0.02)	<0.01	-0.07 (-0.1, -0.03)	<0.01	-0.08 (-0.13, -0.03)	<0.01	-0.07 (-0.1, -0.03)	<0.01
PHQ-9≥10	163	-0.57 (-0.95, -0.19)	<0.01	-0.83 (-1.19, -0.47)	<0.01	-0.75 (-1.28, -0.22)	0.01	-0.72 (-1.1, -0.34)	<0.01
**Anxiety symptoms**									
GAD-7 (range 0-21)	163	-0.06 (-0.1, -0.02)	<0.01	-0.06 (-0.1, -0.02)	<0.01	-0.08 (-0.13, -0.02)	<0.01	-0.07 (-0.1, -0.03)	<0.01
GAD-7≥10	163	-0.51 (-0.91, -0.1)	0.01	-0.43 (-0.83, -0.03)	0.03	-0.73 (-1.3, -0.17)	0.01	-0.56 (-0.97, -0.15)	0.01
**Nighttime symptoms**									
PSQI (range 0-21)	112	-0.01 (-0.08, 0.05)	0.67	-0.04 (-0.11, 0.03)	0.27	-0.05 (-0.14, 0.04)	0.29	-0.03 (-0.1, 0.03)	0.32
PSQI > 5	112	-0.13 (-0.55, 0.3)	0.56	-0.22 (-0.66, 0.21)	0.31	-0.01 (-0.59, 0.57)	0.98	-0.12 (-0.55, 0.31)	0.58
**Insomnia symptoms**									
Athens Insomnia Scale Score (range 0-24)	158	-0.05 (-0.08, -0.01)	0.01	-0.05 (-0.08, -0.01)	0.01	-0.07 (-0.12, -0.02)	0.01	-0.06 (-0.09, -0.02)	<0.01
Athens Insomnia Scale Score≥6	158	-0.53 (-0.87, -0.19)	<0.01	-0.48 (-0.82, -0.15)	0.01	-0.77 (-1.24, -0.3)	<0.01	-0.59 (-0.94, -0.25)	<0.01

Effect estimates are expressed for a 1-unit increase in each of the continuous scales

All models are adjusted for participants' age, sex, education and marital status, obesity (BMI≥30kg/m^2), hypertension, CVD, diabetes, hyperlipidemia, Obstructive Sleep Apnea, osteoporosis, cancer, depression and anxiety disorder.

Table S2. Adjusted associations between perceived low social support (binary variable, high support was set as the referent category) and PROMs, estimated by logistic regression models.

Symptoms	N	MSPSS “significant other” ≤5 N=92 (54.8%)		MSPSS “family” ≤5 N=73 (43.5%)		MSPSS “friends” ≤5 N=109 (64.9%)		MSPSS total ≤5 N=114 (67.9%)	
		OR (95%CI)	p-value	OR (95%CI)	p-value	OR (95%CI)	p-value	OR (95%CI)	p-value
**CAT score**	163	1.16 (1.08, 1.25)	<0.01	1.08 (1.01, 1.15)	0.02	1.12 (1.03, 1.2)	0.01	1.2 (1.09, 1.31)	<0.01
CAT score≥10	163	4.52 (1.99, 10.24)	<0.01	3.43 (1.47, 7.97)	<0.01	3.76 (1.6, 8.8)	<0.01	6.15 (2.47, 15.3)	<0.01
**Daytime symptoms**									
**Fatigue**									
FSS (range 9-63)	161	1.04 (1.01, 1.06)	0.01	1.04 (1.01, 1.07)	0.01	1.06 (1.03, 1.1)	<0.01	1.07 (1.03, 1.11)	<0.01
FSS ≥ 36	161	1.59 (0.76, 3.32)	0.22	2.04 (0.94, 4.46)	0.07	2.49 (1.11, 5.6)	0.03	3.21 (1.37, 7.5)	0.01
**Daytime sleepiness**									
ESS (range 0-24)	160	1.1 (1.01, 1.19)	0.03	1.11 (1.03, 1.22)	0.01	1.12 (1.02, 1.24)	0.02	1.1 (1, 1.22)	0.05
ESS≥11	160	1.84 (0.66, 5.16)	0.24	2.94 (1.05, 8.19)	0.04	2.45 (0.73, 8.19)	0.15	2.04 (0.61, 6.85)	0.25
**Depressive symptoms**									
PHQ-9 (range 0-27)	163	1.12 (1.02, 1.23)	0.01	1.15 (1.05, 1.26)	<0.01	1.17 (1.04, 1.31)	0.01	1.19 (1.07, 1.34)	<0.01
PHQ-9≥10	163	1.89 (0.71, 5.03)	0.2	3.35 (1.25, 9.03)	0.02	1.89 (0.61, 5.82)	0.27	3.97 (1.02, 15.39)	0.05
**Anxiety symptoms**									
GAD-7 (range 0-21)	163	1.12 (1.02, 1.23)	0.02	1.14 (1.03, 1.26)	0.01	1.13 (1.01, 1.25)	0.03	1.11 (1, 1.23)	0.05
GAD-7≥10	163	2.11 (0.77, 5.77)	0.14	3.05 (1.1, 8.41)	0.03	3.21 (0.92, 11.22)	0.07	3.28 (0.93, 11.61)	0.07
**Nighttime symptoms**									
PSQI (range 0-21)	112	1.09 (0.92, 1.28)	0.31	1.11 (0.95, 1.29)	0.2	1.24 (1, 1.53)	0.05	1.27 (1.02, 1.58)	0.03
PSQI > 5	112	1.19 (0.43, 3.29)	0.74	1.14 (0.43, 3.04)	0.79	1.82 (0.58, 5.71)	0.3	2.3 (0.74, 7.22)	0.15
**Insomnia symptoms**									
Athens Insomnia Scale Score (range 0-24)	158	1.19 (1.08, 1.31)	<0.01	1.15 (1.04, 1.26)	0.01	1.22 (1.09, 1.36)	<0.01	1.27 (1.12, 1.43)	<0.01
Athens Insomnia Scale Score≥6	158	5.31 (2.11, 13.36)	<0.01	5.28 (1.87, 14.89)	<0.01	5.95 (2.3, 15.4)	<0.01	6.52 (2.48, 17.11)	<0.01

Adjusted associations between perceived low social support (binary variable, high support was set as the referent category) and daytime and nighttime symptom, estimated by logistic regression models.

Effect estimates are expressed for a 1-unit increase in each of the continuous scales

All models are adjusted for participants' age, sex, education and marital status, obesity (BMI≥30kg/m^2), hypertension, CVD, diabetes, hyperlipidemia, Obstructive Sleep Apnea, osteoporosis, cancer, depression and anxiety disorder.
